# IFCC recommendation on sampling, transport and storage for the determination of the concentration of ionized calcium in whole blood, plasma and serum

**DOI:** 10.1155/S1463924691000391

**Published:** 1991

**Authors:** A. B. T. J. Boink, B. M. Buckley, T. F. Christiansen, A. K. Covington, A. H. J. Maas, O. Müller-Plathe, Ch. Sachs, O. Siggaard-Andersen

**Affiliations:** 1 Zwaardemakerlaan 45 Utrecht 3571 ZB The Netherlands

## Abstract

The substance concentration of ionized calcium (*c*_Ca_^2+^) in blood, plasma or serum preanalytically may be affected by pH changes of the sample, calcium binding by heparin, and dilution by the anticoagulant solution.

pH changes in whole blood can be minimized by anaerobic sampling to avoid loss of Co_2_, by measuring as soon as possible, or by storing the sample in iced water to avoid lactic acid formation. *c*_Ca_^2+^ and pH should be determined simultaneously.

Plasma or serum: If centrifuged in a closed tube, and measured immediately, the pH of the sample will be close to the original value. If a delay has occurred between centrifugation and the measurement, causing substantial loss of Co_2_, equilibration of the sample with a gas mixture corresponding to pCO2= 5.3 kPa prior to the measurement is recommended. Conversion of the measured values to *c*_Ca_^2+^ (7.4) is only valid if the pH is in the range 7.2-7.6.

Ca^2+^ binding by heparin can be minimized by using either of the following:

(1) A final concentration of sodium or lithium heparinate of 15 IU/ml blood or less

(2) Calcium titrated heparin with a final concentration of less than 50 IU/ml blood.

Dilution effect can be avoided by use of dry heparin in capillaries or syringes. When heparin solutions are used, errors due to dilution or calcium binding can be reduced by using syringes with a heparin solution containing free calcium ions corresponding to the mean concentration of ionized calcium in normal plasma.

Conditions for blood collection, storage, and transport to avoid preanalytical errors are described in this paper.


Journal of Automatic Chemistry, Vol. 13, No. 5 (September–October 1991), pp. 235–239

*International Federation of Clinical Chemistry, Scientific Division*

### IFCC recommendation on sampling, transport and storage for the determination of the concentration of ionized calcium in whole blood, plasma and serum

**A. B. T. J. Boink (NL), B. M. Buckley (GB), T. F. Christiansen (DK), A. K. Covington (GB), A. H. J. Maas (NL), O. Müller-Plathe (DE), Ch. Sachs (FR) and O. Siggaard-Andersen (DK)**

*The substance concentration of ionized calcium ($c_{Ca^{2+}}$) in blood, plasma or serum preanalytically may be affected by pH changes of the sample, calcium binding by heparin, and dilution by the anticoagulant solution.*

*pH changes in whole blood can be minimized by anaerobic sampling to avoid loss of $CO_2$, by measuring as soon as possible, or by storing the sample in iced water to avoid lactic acid formation. $c_{Ca^{2+}}$ and pH should be determined simultaneously.*

*Plasma or serum: If centrifuged in a closed tube, and measured immediately, the pH of the sample will be close to the original value. If a delay has occurred between centrifugation and the measurement, causing substantial loss of $CO_2$, equilibration of the sample with a gas mixture corresponding to $p_{CO_2}$ = 5·3 kPa prior to the measurement is recommended. Conversion of the measured values to $c_{Ca^{2+}}$ (7·4) is only valid if the pH is in the range 7·2–7·6.*

*$Ca^{2+}$ binding by heparin can be minimized by using either of the following:*

*(1) A final concentration of sodium or lithium heparinate of 15 IU/ml blood or less*
*(2) Calcium titrated heparin with a final concentration of less than 50 IU/ml blood.*

*Dilution effect can be avoided by use of dry heparin in capillaries or syringes. When heparin solutions are used, errors due to dilution or calcium binding can be reduced by using syringes with a heparin solution containing free calcium ions corresponding to the mean concentration of ionized calcium in normal plasma.*

*Conditions for blood collection, storage , and transport to avoid preanalytical errors are described in this paper.*

### Introduction

The calcium fractions in plasma are in equilibrium and include ionized calcium (free calcium) and bound calcium (complex-bound calcium and protein-bound calcium). After sampling, the substance concentration or ionized calcium is mainly influenced by pH and such ligands as bicarbonate, lactate, phosphate and anticoagulants.

For clinical interpretation ionized calcium concentration and pH should be measured simultaneously [1].

The substance concentration of ionized calcium can be measured in heparinized blood, heparinized plasma or serum. The reported value should refer to the actual pH of anaerobically sampled blood ($c_{Ca^{2+}}$(act)) because this most closely reflects the *in vivo* ionized calcium activity in plasma.

Besides the actual $c_{Ca^{2+}}$, the ionized calcium concentration adjusted to a pH of 7·4 can be calculated ($c_{Ca^{2+}}$(7·4)). This eliminates the influence of $p_{CO_2}$ induced alterations of the pH on the concentration of ionized calcium *in vivo* and *in vitro*. It should be emphasized that $c_{Ca^{2+}}$(7·4) is calculated on the basis of the relationship between $c_{Ca^{2+}}$ and pH obtained when $p_{CO_2}$ varies *in vitro*. The relationship is almost the same for acute $p_{CO_2}$ changes *in vivo*. Little is known about the optimal values of $c_{Ca^{2+}}$ or $c_{Ca^{2+}}$(7·4) in patients with chronic acidemia or alkalaemia.

The calculation of $c_{Ca^{2+}}$(7·4) can compensate for the influence of preanalytical pH alterations on ionized calcium results. For separated plasma or serum, $c_{Ca^{2+}}$(7·4) should be used, because plasma or serum, even when handled anaerobically, tend to give erroneously high pH values unless separated at 37 °C [2].

The determination of $c_{Ca^{2+}}$ is affected by specific preanalytical factors which may induce changes in the final result of the same order of magnitude as physiological or pathological variations. The main factors are:

(1) Influences of pH on the ionized calcium concentration.
(2) Calcium binding by the anticoagulant.
(3) Sample dilution by the anticoagulant solution.

In addition, some special aspects of collection, storage, and choice of sampling technique with regard to the clinical situation have to be considered.

Correspondence should be addressed to Professor Dr A. H. J. Maas, Zwaardemakerlaan 45, 3571 ZB Utrecht, The Netherlands.

© IFCC 1991

### Avoidance of pH changes

The main causes of pH changes *in vitro* are loss of $CO_2$ by non-anaerobic handling and formation of lactic acid by glycolysis in red cells and leukocytes.

*Whole blood samples in syringes or capillaries*

Loss of $CO_2$ must be minimized by applying the rules of anaerobic sampling as used in blood gas analysis. Small amounts of $CO_2$ are absorbed by some plastic materials, leading to a gradual increase of pH. Formation of lactic acid can be avoided by minimizing the delay before measurement (<15 min). If storage is unavoidable, syringes and capillaries should be kept in iced water. Of course, the ionized calcium concentration is valid only if pH alterations have been avoided.

*Plasma or serum samples in tubes*

The tube should be completely filled with blood and should remain closed during centrifugation and throughout processing until the measurement is performed. After centrifugation, serum or plasma should be sampled by aspiration from a layer just above the cells [3]. Pouring the liquid from one tube to another is not suitable [4].

To minimize the effect of lactic acid formation, serum or plasma should be separated from erythrocytes within 1 h. An increase in pH above 7·9 may lead to a decrease of $c_{Ca^{2+}}$ by precipitation of calcium phosphate, especially if elevated phosphate levels are present, as in haemodialyzed patients [5], or by formation of calcium carbonate.

If anaerobic handling is impossible, serum or plasma must be equilibrated immediately prior to measurement with a gas mixture containing 5·7% $CO_2$ corresponding to $p_{CO_2}$ = 5·3 kPa (these figures are valid for equilibration at 37 °C; equilibration at 25 °C requires a gas mixture containing: 3·2% $CO_2$). The pH induced by this procedure should be in the range 7·2–7·6, otherwise the conversion of the measured ionized calcium concentration to pH 7·4 is invalid [6].

*pH dependent transformation of ionized calcium results*

The measured concentration of ionized calcium may be converted to pH = 7·40 by equation (1) [6]:

$$\lg c_{Ca^{2+}}(7{\cdot}4) = \lg c_{Ca^{2+}}(x) - S\,(7{\cdot}40\text{-pH}(x)) \quad (1)$$

where S is a slope factor and x indicates the measured values. Consequently, the concentration of ionized calcium at the actual pH may be estimated from the converted value by equation (2) for example if the actual pH has been measured independently.

$$\lg c_{Ca^{2+}}(\text{act}) = \lg c_{Ca^{2+}}(7{\cdot}4) + S\,(7{\cdot}40\text{-pH(act)}) \quad (2)$$

S was originally determined as 0·24 for serum and 0·22 for whole blood [1]. Values of S between 0·16 and 0·20 have been reported with different instruments [7,8]. The values were determined by equilibration with different $CO_2$ mixtures. The slope differs if pH is changed by HCl or lactic acid [1], or when the albumin concentration is abnormal [9].

Because of the abundance of influences causing different slopes, and because the relation between lg $c_{Ca^{2+}}$ and pH is approximately linear only for a limited pH interval around 7·4, these conversions have to be limited to the pH interval 7·2 to 7·6 of samples with normal albumin/total protein levels.

### Avoidance of $Ca^{2+}$-binding by the anticoagulant

At present, the only adequate anticoagulant for ionized calcium determinations is heparin. Heparin is known to bind small but significant amounts of calcium to an extent which depends on the quantity and type of heparin being used [7].

*Calcium titrated heparinate* is the best available means to minimize calcium binding. The heparinate should be titrated to an ionized calcium concentration corresponding to the midpoint of the reference range in plasma. The original $c_{Ca^{2+}}$ of the sample will then be modified proportionally to the difference between its value and the titration value. These changes, however, are negligible if the final concentration of calcium titrated heparin is below 50 IU/ml blood [4].

If *heparin preparation with calcium titration* are used the final heparin concentration should not be higher than 15 IU/ml blood. In this case the extent of calcium binding is small and will not exceed 2%.

To ensure proper anticoagulation, careful mixing immediately after sampling is necessary, especially when the anticoagulant is in a dry state. Mixing is achieved by rolling syringes between the hand, by moving a mixing wire along the full length of a capillary tube with a magnet, or by inverting tubes repeatedly.

*Syringes*

Filling a 5 ml syringe with blood up to the nominal volume and assuming a dead space of 0·2 ml containing calcium-titrated heparin (1000 IU/ml), a final concentration of 40 IU/ml can be expected. (Of course syringes should always be filled to the specified volume, see section 4.)

*Capillary tubes*

To ensure proper anticoagulation in capillary tubes, higher concentrations of dry heparinate are required. However, the heparinate concentration should not exceed 50 IU/ml. With such high concentrations it is necessary to use calcium titrated heparinate.

*Tubes*

It should be noted that the final concentration of heparin should not exceed 15 IU/ml of blood.

Constituents like fluid separators or clotting activators may only be used if they do not influence the concentration or the measurement of ionized calcium [10,11].

### Avoidance of dilution effects caused by anticoagulant solutions

Sample dilution by anticoagulant solutions may occur particularly when syringes are used for sampling, or, less commonly, when heparinate solution is added to collection tubes. Glass and plastic syringes are often used for anaerobic blood sampling. The dead space of the syringe and the needle is filled with a heparin solution containing free calacium ions to an extent corresponding to the midpoint of the reference range for ionized calcium in plasma. Alterations in the original concentration of ionized calcium can thus be minimized.

The dead space of a 10 ml or 5 ml syringe is 2 to 6% when the syringe is filled to their nominal volume. With 2 ml or 1 ml syringes, the dilutions may be even greater, and the effect is enhanced if syringes are filled with less then their nominal volume [4,12,13]. Disposable syringes containing dry calcium titrated heparinate are preferable.

### Site of blood collection

$c_{Ca^{2+}}$ can be measured in arterial capillary and venous blood. Arterial and capillary sampling requires the same precautions as for pH and blood gas analysis. For venous sampling, it is important to avoid factors modifying the pH value of the specimen. Venous sampling should preferably be done without a tourniquet. If the use of a tourniquet is unavoidable, sampling should be completed with 2 min. Stasis and muscular action like 'pumping' cause changes of the ionized calcium concentration [5,14].

### Storage and transport

*Storage of whole blood samples*

$c_{Ca^{2+}}$(act) and pH(act) should be measured within 15 min in samples kept at room temperature, and within 4 h in samples stored at 4 °C. $c_{Ca^{2+}}(7{\cdot}4)$ can be determined within 6 h in samples kept at room temperature, and within 24 h in samples stored at 4 °C [5].

*Storage of serum or plasma samples*

In separated plasma or serum, $c_{Ca^{2+}}(7{\cdot}4)$ should be determined (see section 1). Plasma or serum can be stored in plain glass tubes at 4 °C for 1 week and at −20 °C for 6 weeks without significant changes in $c_{Ca^{2+}}(7{\cdot}4)$, whereas a significant fall (−2·4%) is observed after 4 months [5].

*Transport conditions*

Only separated serum or plasma kept in closed tubes should be used for transport exceeding 4 hours. The samples may be transported at ambient temperature, but they should be stored at 4 °C. The samples should be processed within 7 days with measurement of $c_{Ca^{2+}}$ and pH after preceding $CO_2$-equilibration (see section 2.2) and subsequent calculation of $c_{Ca^{2+}}(7{\cdot}4)$. Valid results for the actual pH and the actual ionized calcium concentration of the patient cannot be expected if samples have been mailed.

### Choice of sampling technique with regard to the clinical situation

*Routine*

$c_{Ca^{2+}}$ determinations can usually be performed either in anaerobically handled whole blood samples, or in venous plasma or serum samples handled as described in the preceding sections. If only $c_{Ca^{2+}}(7{\cdot}4)$ could be determined, and if the patient's pH has been measured independently, $c_{Ca^{2+}}$(act) can be estimated by calculation according to Section 2.3.

In non-emergency situations, preanalytical factors like venous stasis, position, nutritive state and diurnal variation have to be respected. If possible, the blood collection should be done between 8 and 10 a.m. on the fasting subject in a supine position using the sampling technique as described in section 5.

*Urgent situations*

In urgent situations (for example requests from intensive care units, haemodialysis, cardiovascular surgery) the results have to be reported as quickly as possible. This is ensured by working with strictly anaerobically handled whole blood samples collected in syringes or capillaries which require no further sample preparation. In these cases, both the actual ionized calcium concentration and pH should be reported.

*Paediatrics*

Because of the limited sample volume, sampling should be done anaerobically in capillary tubes or 1 ml syringes containing calcium titrated heparinate in a dry state (see sections 2.1 and 3.2). The syringe should be filled to its nominal volume. The sample has to be mixed carefully, avoiding haemolysis. The measurement should be performed on whole blood. This procedure minimizes the effects of sample dilution, calcium binding, and $CO_2$ loss.

*Establishing reference intervals*

From the preceding paragraphs, it is clear that, to define reference intervals, samples must be collected and handled under strictly standardized preanalytical conditions. Selection and partition factors, kind of specimen (blood, plasma, serum) and site of collection have to be taken into account.

### Reports of results

The laboratory report on $c_{Ca^{2+}}$ should contain the following additional information:

(1) Type of sample: blood, plasma or serum.
(2) Site of collection: arterial, capillary or venous.
(3) Kind of quantity: actual substance concentration of ionized calcium, substance concentration of ionized calcium converted to a pH of 7·4 and actual pH.

*Table 1. Types of specimen for determination of* $c_{Ca^{2+}}$.

| Specimen | $Ca^{2+}$-binding by anticoagulant | Dilution by anticoagulant | Handling | Limitations |
|---|---|---|---|---|
| Whole blood, liquid sodium or lithium heparinate. Whole blood liquid Ca-titrated heparin. | Below 2% as long as heparin conc. is below 15 IU/ml blood. Negligible for specimens with normal $c_{Ca^{2+}}$. | Depending upon the volume of heparin solution in a tube or the relative dead space of a syringe, respectively. In many cases the dead space is about 5% of the nominal volume. | 1. Pretreated sampling containers necessary, normally syringes or capillaries. 2. Anaerobic sampling necessary, arterial, venous or by skin procedures. 3. To be stored at 4 °C if not measured within 15 min. | If the specimen is sampled and handled anaerobically and stored appropriately, and albumin and total protein are normal, $c_{Ca^{2+}}$(act) and pH(act) can be measured accurately and $c_{Ca^{2+}}$(7·4) can be calculated (limited pH range). |
| Whole blood, dry sodium or lithium heparinate. | Approx. 5% with a heparin conc. of 50 IU/ml blood, which is regarded as necessary for safe anti-coagulation with a dry preparation. | None | | |
| Whole blood, dry Ca-titrated heparin. | Negligible for specimens with normal $c_{Ca^{2+}}$. | None | | |
| Serum | None | None | 1. Sampling and sample handling routine avoiding $CO_2$ losses as far as possible. 2. Pre-equilibration recommended if $CO_2$ loss may have occurred. | Preferably $c_{Ca^{2+}}$(7·4) should be reported. The validity of this quantity is limited in presence of acid-base disorders and/or in situations where the serum proteins, especially albumin are significantly abnormal. |

### References

1. Thode, J., Fogh-Andersen, N., Wimberley, P. D., Moller-Sorensen, A. and Siggaard-Andersen, O., Relation between pH and ionized calcium *in vitro* and *in vivo* in man. *Scandinavian Journal of Clinical Laboratory Investigation*, **43**, Suppl. 165 (1983), 79–82.
2. Siggaard-Andersen, O., *The Acid-Base Status of the Blood*, 4th revised edition. Munksgaard, Copenhagen (1974), 149–154.
3. Graham, G. A. and Johnson, L. A., The stability of serum ionized calcium during sampling handling and analysis. In: *Methodology and Clinical Applications of Ion-Selective Electrodes*. Vol. 10, Maas, A. H. J., Buckley, B, Manzoni, A., Moran, R. F., Siggaard-Andersen, O. and Sprokholt, R. (Eds). Elinkwijk, Utrecht, The Netherlands (1979), 55.
4. Sachs, Ch. and Thode, J., Preanalytical errors in ionized calcium determination in blood: A review for a subsequent recommendation for blood sampling. In: *Methodology and Clinical Applications of Ion Selective Electrodes*, Vol. 7, Maas, A. H. J., Boink, A. B. T. J., Saris, N.-E. L., Sprokholt, R. and Wimberley, P. D. (Eds). Radiometer, Copenhagen (1986), 259–270.
5. Thode, J., Fogh-Anderson, N., Aas, F. and Siggaard-Andersen, O., Sampling and storage of blood determination of ionized calcium. *Scandinavian Journal of Clinical Laboratory Investigation*, **45** (1985), 131–138.
6. Siggaard-Andersen, O., Thode, J. and Wandrup, J., The concentration of free calcium ions in the blood plasma: 'ionized calcium'. In: *Blood pH, Carbon Dioxide, Oxygen and Calcium-ion*. Siggaard-Andersen, O. (Ed.). Private Press, Copenhagen (1981), 163–190.
7. Sachs, Ch., Chaneac, M., Rabouine, Ph. and Kindermans, C., Effect of calcium ligands on the pH standardization of ionized calcium measurements in blood: a theoretical approach. In: *Methodology and Clinical Applications of Ion-Selective Electrodes*. Vol. 9, Burritt, M. F., Cormier, A. D., Maas, A. H. J., Moran, R. F. and O'Connell, K. M. (Eds). Davies Printing Company, Rochester (1988), 228–233.
8. Rawson, K. and Verghese, D. A. Personal communication.
9. Buckley, B. M., Rawson, K., Russel, L. J. and Smith, S. C. H., The influence of protein concentration on the mathematical adjustment of ionized calcium measurements for pH. In: *Methodology and Clinical Applications of Ion-Selective Electrodes*. Vol. 9, Burritt, M. F., Cormier, A. D., Maas, A. H. J., Moran, R. F. and O'Connell, K. M. (Eds). Davies Printing Company, Rochester (1988), 121–130.
10. Larsson L. and Ohman St., Effect of silicon-separator tubes and storage time on ionized calcium in serum. *Clinical Chemistry*, **31** (1985), 169–170.
11. Toffaletti, J., Blosser, N. and Kirvan, K., Effects of storage temperature and time before centrifugation on ionized calcium in blood collected in plain tubes and silicon-separator (SST) Tubes. *Clinical Chemistry*, **30** (1984), 553–556.
12. Sachs, Ch., Rabouine, Ph., Kindermans, C. and Sage, C. Preanalytical dilution errors in plasma and whole blood ionized calcium determinations on small sized samples. In: *Methodology and Clinical Applications of Ion-Selective Electrodes*, Vol. 8, Maas, A. H. J., Buckley, B., Marsoner, H., Saris, N.-E. L. and Sprokholt, R. (Eds). Radiometer, Copenhagen (1987), 117–123.

13. Sachs, Ch., Rabouine, Ph., Chaneac, M. and Kindermans, C. Preanalytical dilution errors in ionized calcium on plasma and whole blood. Part II. Effect of specimen volume to syringe nominal volume ratio. In: *Methodology and Clinical Applications of Ion-Selective Electrodes*, Vol. 9, Burritt, M. F., Cormier, A. D., Maas, A. H. J., Moran, R. F. and O'Connell, K. M. (Eds). Davies Printing Company, Rochester (1988), 108–113.
14. Renoe, B. W., McDonald, J. M. and Ladenson, J. H., The effect of stasis with and without exercise on free calcium, various cations and related parameters. *Clinical Chimica Acta*, **103** (1980), 91–100.

